# Soccer pass performance following caffeine intake with deliberate or maintenance practice

**DOI:** 10.1080/15502783.2026.2663140

**Published:** 2026-04-21

**Authors:** Burak Çağlar Yaşlı, Özcan Esen, Aysberg Şamil Önlü, Oğuzhan Tuncel, Muhammed Uygar Sertkaya, Alejandro Muñoz, Raci Karayiğit

**Affiliations:** a Department of Coaching Education, Iğdır University, Iğdır, Türkiye; b Department of Sport, Exercise & Rehabilitation, Faculty of Health and Life Sciences, Northumbria University, Newcastle upon-Tyne, UK; c Department of Sport Management, Lokman Hekim University, Ankara, Türkiye; d Institute of Health Sciences, Ankara University, Ankara, Türkiye; e Exercise and Sport Science, Universidad Francisco de Vitoria, Madrid, Spain; f Department of Coaching Education, Ankara University, Ankara, Türkiye

**Keywords:** Sports nutrition, ergogenic aid, team sports, passing, skill

## Abstract

**Background:**

The impact of caffeine on strength and endurance performance is well acknowledged, yet its influence on skill performance remains contentious. A potential scenario in which caffeine augments the efficacy of practice could be useful for sports brokers who diligently pursue every nuance to enhance performance. Therefore, the primary objective of this study was to examine the impact of 3 mg·kg^−1^ of caffeinated coffee intake combined with deliberate (DP) or maintenance practice (MP) on passing performance in adolescent football players. The study also discusses how DP and MP affect passing accuracy without considering caffeine or placebo conditions, as well as how athletes perceive DP and MP and whether caffeine supplementation influences these perceptions.

**Methods:**

Fourteen adolescent male football players (14.07 ± 0.26 years; 174.28 ± 3.12 cm; 57.21 ± 8.40 kg) participated in a double-blind, randomized, counterbalanced, and crossover research design. For the experimental protocols, each participant visited an artificial turf pitch on four occasions, separated by 48 h. They received 3 mg·kg^−1^ of caffeine sourced from coffee with DP (1), caffeinated coffee intake with MP (2), decaffeinated coffee with DP (3), and decaffeinated coffee with MP (4). Upon concluding the practice regimes, the athletes promptly expressed their evaluations of the practice on a scale of 1–10. The Loughborough Soccer Passing Test (LSPT), the One-Touch Passing Test (OTPT), and the Long Passing Test (LPT) were administered to evaluate participants' passing proficiency at both the beginning and end of each session.

**Results:**

There was no difference in LSPT, OTPT, or LPT values following caffeine (CAF) and placebo (PLA) supplementation after DP or MP. Regardless of CAF-PLA conditions, although both practices improve the LSPT original time, penalty time, and performance time, only MP increases the LPT score (21.9%; *p* = 0.03). Caffeine also has no additional modifier effect on practice perceptions. DP is considered more mentally challenging than MP (4.18 ± 2.3 & 1.9 ± 1.2; *p* > 0.05), but both practices are similar in terms of relevancy, enjoyment, and physicality.

**Conclusion:**

3 mg·kg^−1^ of caffeinated coffee has no additional effects on DP or MP for passing performance. Regardless of CAF or PLA intake, both practices improve short-term passing, yet only MP appears effective for enhancing long-term passing in players with average technical ability. Accordingly, coaches may consider incorporating these strategies into pre-match warm-ups or structured training programs. Moreover, CAF did not influence players’ perceptions of the training sessions, particularly when physical demand was minimal. Similarly, when comparing DP and MP, athletes reported similar perceptions, suggesting that the practical application of DP in field-based settings may diverge from its original theoretical framework. Further research needs to clarify how DP principles are implemented and perceived in real-world practice.

## 1. Introduction

Coffee, which contains caffeine, is the second most widely consumed beverage after water, with an estimated global consumption of over 2 billion cups per day [1]. Athletes frequently consume it to enhance physical and cognitive performance [2]. According to the International Society of Sports Nutrition, coffee is a complex matrix of hundreds of bioactive compounds – not just caffeine – whose neuromuscular, cognitive, and metabolic effects may contribute to enhanced exercise performance under certain conditions [3]. Research indicates that a dosage of 3 mg/kg of caffeine, administered 45–60 min before exercise, enhances athletic performance. This effect is likely due to adenosine A_1_ and A_2A_ receptor antagonism and the activation of the NA+ /K+  ATPase pump [4]. Astorino et al. [4]. proposed that the ergogenic mechanisms of caffeine are likely multifactorial, given the observed enhancements in reaction time, cognition, and mood following caffeine consumption, which may contribute to soccer skill performance [5]. In line with this, Mielgo-Ayuso et al. [6] reported that a moderate dose of caffeine consumed before soccer activity may enhance physical performance variables such as jump height, repeated sprint ability, and running distance, without increasing muscle damage or perceived fatigue; however, this study did not assess football-specific technical skills.

Passing is the most prevalent technical ball action in football [7]. Approximately one thousand passes – over ten per minute – occur in a typical match, with the majority being short passes, while long passes constitute only approximately 10% of the total [8–11]. Although passing ability alone does not guarantee player- or team-level success in football [8,12–15], Dellal et al. [16]. emphasized that elite-level football considers a pass accuracy of at least 70% as a minimum standard.

Practice makes perfect, but there has been a historical debate over which method is more effective [17–19]. In their far-famed study, Ericsson et al. [18]. discovered that the amount of deliberate practice (DP), not other forms of practice, differentiates the most exceptional people from their good or average counterparts. For sure, the most effective learning practice needs to be deliberately structured and identify weaknesses rather than the mere repetition of tasks [19–21]. Since the day of its establishment, DP has had many criticisms, and numerous studies have reported inconsistencies regarding its efficacy in sport [22–25]. Rather, the prevailing practice activities of training environments in modern sport mainly include maintenance (MP) or other forms of activities such as play and purposeful drills [26–28].

A potential avenue of short-term exchanges for passing proficiency is the implementation of nutritional ergogenic aid intake [29,30]. Caffeine is one of the most extensively investigated ergogenic aids and has been shown to enhance mental alertness and aspects of cognitive function, which may support task accuracy; however, caffeine consumption may also induce tremors or agitation, potentially impairing movement accuracy [30]. Jafari et al. [31] investigated the effect of 3 mg·kg^−1^ of caffeine ingestion on decision-making and passing accuracy in young male soccer players. No clear differences were observed between the caffeine and placebo conditions for short- or long-passing performance. By comparison, Fosket et al. [32] found that 6 mg·kg^−1^ of caffeine ingestion enhances passing accuracy during soccer-simulated activity, in contrast to Andrade Souza et al [33]. and Shabir et al. [34], who demonstrated that caffeine or caffeine with carbohydrates has no additional effect on passing performance in amateur and recreational soccer players. Although these studies raise questions regarding the ergogenic effect of caffeine on skill performance, no research has yet investigated the ergogenic effect of caffeine on skill performance in conjunction with any domain-specific practice. Furthermore, two recent extensive reviews [5,29] reported that the ergogenic effect of caffeine on skill performance in team sports is limited and unclear. More research is warranted.

Therefore, the primary objective of this study is to examine how 3 mg·kg^−1^ of caffeinated coffee intake combined with DP or MP impacts pass prowess in adolescent football players. The study also discusses how DP and MP affect passing accuracy without considering caffeine or placebo consumption as well as how athletes perceive DP and MP and whether caffeine supplementation influences these perceptions.

## 2. Materials and methods

### 2.1. Participants

Fourteen adolescent male football players participated in the study (14.07 ± 0.26 years; 174.28 ± 3.12  cm; 57.21 ± 8.40 kg; football experience = 5.35 ± 1.82). All players are affiliated with the same club academy that competes in the Turkish national youth championships. They are trained regularly three days a week (3 × 75 min.). Only adolescent male football players between 13 and 15 years of age were eligible for inclusion. The exclusion criteria included caffeine intolerance, the presence of any chronic disease or injury in the month prior to the study, habitual caffeine consumption greater than 100 mg·day⁻¹ (as assessed by a food frequency questionnaire), while some studies suggest that habitual consumption may not substantially influence the ergogenic effect of caffeine [35], and the use of medications or dietary supplements during the study period. Written informed consent was obtained from players and their parents prior to the commencement of the study. The study procedures adhered to the principles established in the Declaration of Helsinki and received approval from the Ankara University Ethical Committee (10-784-24).

### 2.2. Study design

**Figure 1.**
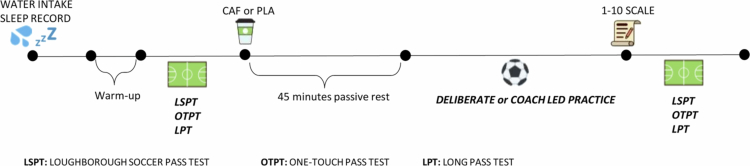
Experimental protocol.

A double-blind, randomized, counterbalanced, and crossover research design was used in this study. After a familiarization session one week prior to the study, each participant visited an artificial turf pitch on four occasions, separated by 48 h. They intake of 3 mg·kg^−1^of caffeine sourced from coffee with DP (1), caffeinated coffee intake with MP (2), decaffeinated coffee with DP (3), and decaffeinated coffee with MP (4). This 48-h interval was implemented to allow sufficient wash-out of caffeine and minimize any carryover effects between conditions. On test days, following a standard 10-min warm-up protocol, players performed the Loughborough Soccer Passing Test (LSPT), the One-Touch Passing Test (OTPT), and the Long Passing Technique Test (LPT), respectively. Upon completing the tests, they immediately consumed caffeine (CAF) or a placebo (PLA) and were permitted 45 min of passive rest in a seated position, after which they engaged in DP or MP training as appropriate. After completing the practices, players rated their physical effort, mental strain, enjoyment, and usefulness of the exercises on a scale from 1 to 10 [28]; then, they repeated the tests in the same order to complete the session. Figure 1 illustrates a schematic representation of the experimental protocol.

All test sessions took place in the early morning hours (9 a.m.–12 p.m.) under fasting conditions and were performed prior to the teams’ training on the same day to ensure adequate recovery for the subsequent trial. The participants were instructed to avoid caffeine, alcohol ingestion, and strenuous exercise for 24 h prior to the testing sessions. Each player was administered 500 ml of water to mitigate hydration effects prior to the tests. Sleep states are recorded on session day based on self-reported data (8 h for CAF with DP sessions; 8h13min for CAF with MP sessions; 8h16min PLA with DP sessions; 8h29min PLA with MP sessions), and players are informed to report any potential gastrointestinal discomforts during or after the tests (on 5 of 56 occasions, participants reported a sour stomach on a visit day). Habitual caffeine consumption was overseen by a qualified nutritionist and evaluated using a modified version of the caffeine consumption questionnaire developed by Bühler et al. [36]. All participants were naive consumers (<50 mg/day). Caffeinated and decaffeinated (as a placebo) coffee were prepared utilizing instant coffee (Nescafé Gold, Nestlé, Turkey). The caffeine content of the caffeinated coffee was determined based on a reference sample obtained from the same batch by the Ankara Food Control Laboratory Directorate, reporting that 1 g of caffeinated coffee contains 36 mg of caffeine. Accordingly, participants ingested 0.083 g·kg^−1^ caffeinated coffee to intake 3 mg·kg^−1^ caffeine. The exact amount of coffee powder was calculated individually based on body mass. For example, a 60 kg participant ingested 5.00 g of coffee powder, providing approximately 180 mg of caffeine. Nescafe decaffeinated coffee was reported to provide 0.17 mg·kg^−1^ of caffeine, indicating that a very low dose is ergogenic. The participants had 10 min to consume the coffee dissolved in 600  mL of hot water (approximately 70 °C) served in a mug [37].

#### 2.2.1. Skill-technique tests

Short passing skill – LSPT: The LSPT has been documented to exhibit good reliability and validity across various levels of groups for assessing short passing ability (CV: 1.8%–17%) [38,39]. It requires a total of 16 rapid football passes to targets executed with minimal errors in accordance with the tester's instructions. The test duration, penalty duration, and total duration (test duration + penalty duration) were recorded as a result of the test. LSPT details have been described in detail in previous studies [38,39].

Short passing technique – OTPT: Short passing technique was measured using a one-touch passing test (CV: 11.3%) [40]. Athletes received a total of 15 passes from both the right and left sides and tried to hit a mini-goal (1 m × 40 cm) with a single pass that was located 10 m away. The same protocol is applied to both feet with a 3-min interval; they receive one point for each successful hit in the goal, allowing a maximum score of 30 points from the tests.

Long passing technique – LPT: The protocol established by Rostgaard et al. [41] was utilized to evaluate long passing technique (CV: 11.7%–16%). The test comprises 10 long kicks from the passing zone to “the goal zone”, which is positioned 30 m away from the passing zone and includes three nested rectangular areas. A score of 1, 2, or 3 points is given depending on the area where the ball lands for each attempt, and players have to start a new attempt after a 30-second interval. A total of 10 dominant leg attempts are made, and the natural outcome of this one can attain a maximum of 30 points from the test.

### 2.3. DP and MP protocol

To create a deliberate environment, we impose a plethora of Ericsson’s works [18,42–44] in tandem with famed guidelines such as ASPIRE [26] and EXPERTS [20], which elucidate the application of DP in practical contexts. (1) DP is not a trial-and-error process; it is a specific and challenging practice aimed at enhancing areas of deficiency (non-dominant leg). (2) The task is intended to improve individual performance (individual work on pitch). (3) The trainee can complete the task on their own and repeatedly execute the same or similar task (a total of 120 repetitions). (4) The participants need to gain immediate informative feedback, and the task performed aligns with individualized instruction and guidance of a teacher (feedbacks are given in accordance with operational feedback recommendations by Williams and Hodges [45], preferably positive, after trials and if necessary). To modify drills in the DP, common task constraints such as direction of target and player position on the pitch are used [45–47], which align with the following principles of skill training explained by Williams and Hodges [48]. Likewise, practice difficulty is organized according to dual-task scenarios [49,50], establishing optimal challenge points essential for skill acquisition and adaptation [48,51]. For example, the participants had to pass and answer math questions or choose the correct color simultaneously that was announced by the experimenter (Supp. Material). Hodges et al [51]. suggested that medium difficulty is the optimal learning advantage for intermediate learners.

In creating MP, practice classification was determined by Ford et al. [26], and Ericssonn [42] recommendations. Exercises in MP drills were determined after a brief meeting between the team coach and the first author of this article. Traditional passing stations commonly utilized in football training were chosen. Drills in MP are implemented with the author's supervision but without any interferences. This model is based on a traditional approach, prioritizing the execution of the technical component through repetition. Both DP and MP training include three primary passing stations: control short-pass drills, one-touch short-pass drills, and long-pass drills performed in both linear and diagonal directions (Supp. Material). Despite trainees being free to use both legs and not receive feedback in MP, they must use the non-dominant leg at certain times as well as the dominant leg, according to supervisor directives in DP. Deliberate use of a non-preferred foot may reduce lateral asymmetry and improve dominant leg proficiency [40,52]. The total practice durations are 15.28 ± 2.55 and 19.42 ± 1.31 min for DP and MP, which are consistent with previous research [52–54]. To ensure parity between DP and MP loads, repetitions are equalized for both training, which includes 40, 60, and 20 repetitions for control-short passes, one-touch short passes, and long-passing drills, respectively. The techniques were trained in a blocked manner in both practice regimes, but variability inside drills is high in DP compared to MP as a natural outcome of DP structure.

### 2.4. Statistical analysis

All statistical analyses were performed using IBM SPSS Statistics for Windows (version 22.0; IBM Corp., Armonk, NY, USA). Prior to inferential analyses, the data were screened to ensure that the underlying statistical assumptions were met. Baseline equivalence was assessed using paired t-tests. Potential visit (order) effects were examined using repeated-measures ANOVA. A 2 (condition: CAF vs. PLA) × 2 (practice: DP vs. MP) × 2 (time: pre- vs. post-intervention) repeated-measures ANOVA was conducted to examine performance outcomes (LSPT, OTPT, and LPT). Perceptual responses (mental strain, physical effort, enjoyment, and relevancy) were analyzed using a 2 (supplementation: CAF vs. PLA) × 2 (practice: DP vs. MP) repeated-measures ANOVA. When significant main or interaction effects were identified, Bonferroni-adjusted pairwise comparisons were applied. Statistical significance was accepted at *p* < 0.05. Effect sizes were calculated using partial eta squared (η²p), categorized as small (≥0.01), medium (≥0.06), or large (≥0.14). A sensitivity analysis was performed in G*Power (version 3.1.9.4) to evaluate the minimum detectable effect size with the sample size (*N* = 14 participants). The design had sufficient power (0.80) to detect effects of Cohen’s f = 0.263 (equivalent to η²p = 0.065) at *α* = .05.

## 3. Results

Baseline comparisons revealed no significant differences between CAF and PLA conditions or between DP and MP groups for any performance variables (all *p* > 0.05). Additionally, no significant visit effects were observed (all *p* > 0.05).

**Figure 2.**
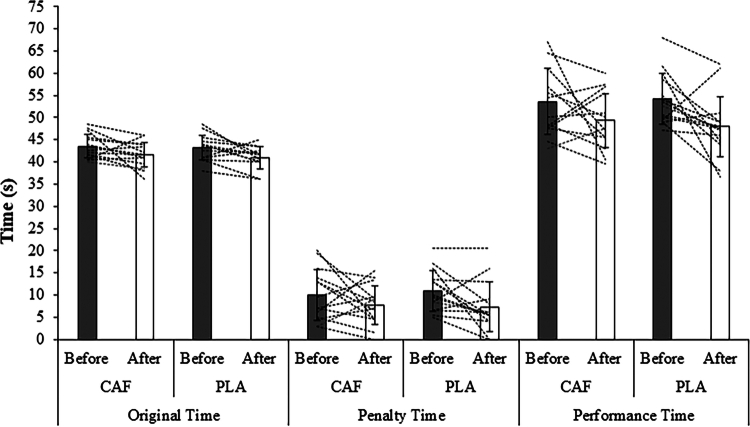
Group mean (SD) and individual responses in on The Loughborough Football Passing Test (LSPT) before and after practice following caffeine (CAF) or placebo (PLA) supplementation are shown in the bars and dashed lines, respectively.

*LSPT:* The group mean LSPT responses in pre- and post-practice following both CAF and PLA supplementation are shown in Figure 2. Table 1 presents pre- and post-test values for LSPT, OTPT, and LPT following DP and MP. A repeated-measures ANOVA revealed no significant main effects of condition for original, penalty, or performance time (F(1, 26) = 0.42, 0.01, and 0.07, respectively; all *p* ≥ 0.521, η²*p* ≤ 0.016). Similarly, no significant main effects of practice were detected (F(1, 26) = 0.51, 2.92, and 0.07, respectively; all *p* ≥ 0.100, η²p ≤ 0.101). In contrast, a significant main effect of time was found for all three variables (F(1, 26) = 9.62, 7.32, and 10.14, respectively; *p* = 0.005, 0.012, and 0.004; η²*p* = 0.270, 0.220, and 0.281), indicating that the original, penalty, and performance times were significantly shorter from pre- to post-intervention. Post-intervention values were lower than pre-intervention by 2.09 s (95% CI −3.47 to −0.71), 3.16 s (95% CI −5.56 to −0.76), and 5.25 s (95% CI −8.64 to −1.86) for the original, penalty, and performance times, respectively. No significant condition × practice (F(1, 26) = 0.30, 2.91, and 2.02; all *p* ≥ 0.100, η²*p* ≤ 0.101), condition × time (F(1, 26) = 0.10, 0.50, and 0.46; all *p* ≥ 0.484, η²*p* ≤ 0.019), or practice × time (F(1, 26) = 0.05, 1.11, and 0.42; all *p* ≥ 0.301, η²*p* ≤ 0.041) interactions were observed. Furthermore, no significant condition × practice × time interactions were detected (F(1, 26) = 0.03, 0.07, and 0.02; all *p* ≥ 0.791, η²*p* ≤ 0.003).

**Table 1.** Effects of deliberate- and maintenance-practice on The Loughborough Football Passing Test (LSPT), One Touch Pass (OTPT), and Long Pass Tests (LPT).

	Deliberate-practice		Maintenance-practice	
LSPT	Before	After	Before	After
*Original time (s)*	43 ± 2	41 ± 2*	44 ± 3	41 ± 2*
*Penalty time (s)*	12 ± 6	7 ± 4*	11 ± 5	8 ± 5*
*Performance time (s)*	55 ± 7	48 ± 5*	53 ± 7	49 ± 6*
				
				
**One Touch Pass (AU)**	15 ± 2	14.1 ± 2.5	15.3 ± 1.9	14.9 ± 1.6
				
**Long Pass (AU)**	10.1 ± 5.1	9.3 ± 4.2	8.2 ± 4.4	10 ± 4.6*
				

^*^ *Indicates significant differences between before and after intervention (p < 0.05).*

**Figure 3.**
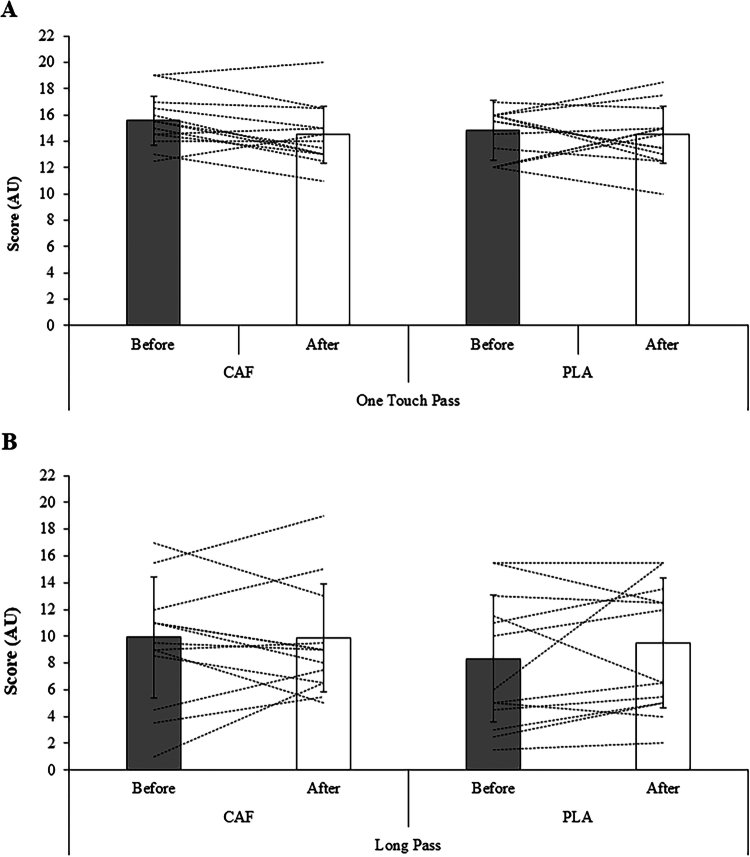
Group mean (SD) and individual responses in on The One Touch Pass (A) and The Long Pass (B) tests before and after practice following caffeine (CAF) or placebo (PLA) supplementation are shown in the bars and dashed lines, respectively.

*OTPT:* The group mean OTPT responses in pre- and post-practice following both CAF and PLA supplementation are shown in Figure 3A. No significant main effect of condition was observed (F(1, 26) = 0.47, *p* = 0.497, η²*p* = 0.018), nor was there a significant main effect of practice (F(1, 26) = 0.56, *p* = 0.461, η²*p* = 0.021). The main effect of time was also not significant (F(1, 26) = 2.21, *p* = 0.148, η²*p* = 0.079). Furthermore, no significant two-way interactions were detected for condition × practice (F(1, 26) = 0.04, *p* = 0.838, η²*p* = 0.002); condition × time (F(1, 26) = 0.83, *p* = 0.370, η²*p* = 0.031); or practice × time (F(1, 26) = 0.67, *p* = 0.420, η²*p* = 0.025). The three-way interaction between condition × practice × time was also not significant (F(1, 26) = 0.67, *p* = 0.419, η²*p* = 0.025).

*LPT:* The group mean LPT responses in pre- and post-practice following both CAF and PLA supplementation are shown in Figure 3B. No significant main effects of condition (F(1, 26) = 1.30, *p* = 0.264, η²*p* = 0.048), practice (F(1, 26) = 0.18, *p* = 0.670, η²*p* = 0.007), or time (F(1, 26) = 0.83, *p* = 0.371, η²*p* = 0.031), on long-pass performance. There were also no significant condition × practice (F(1, 26) = 0.48, *p* = 0.493, η²p = 0.018), condition × time (F(1, 26) = 1.06, *p* = 0.312, η²*p* = 0.039), or three-way condition × practice × time interactions (F(1, 26) = 1.18, *p* = 0.287, η²*p* = 0.043). However, a significant practice × time interaction was detected (F(1, 26) = 4.78, *p* = 0.038, η²*p* = 0.155). Post-hoc comparisons indicated that long-pass scores improved only after MP (mean difference = 1.82 points, 95% CI 0.11–3.53; *p* = 0.038), whereas no significant change was observed following DP (mean difference = −0.75 points, 95% CI −2.46–0.96; *p* = 0.375).

*Perception of DP and MP:* The group mean mental strain, physical effort, enjoyment, and relevancy values in post-practice following DP or MP are reported in Table 2. No significant main effects of supplementation were observed for mental strain, physical effort, enjoyment, or relevancy (F (1, 26) = < 0.01, 1.44, 2.82, and < 0.01, respectively; all *p* ≥ 0.105, η²*p* ≤ 0.098). A significant main effect of practice was found only for mental strain (F (1, 26) = 10.68, *p* = 0.003, η²*p* = 0.291), whereas no significant main effects of practice were detected for physical effort, enjoyment, or relevancy (F(1, 26) = < 0.01, 0.93, 0.01; all *p* ≥ 0.343, η²*p* ≤ 0.035). Post-hoc comparisons indicated that mental strain was significantly higher following DP compared with MP (mean difference = 2.21, 95% CI 0.82–3.61; *p* = 0.003). No significant supplementation × practice interactions were observed for any of the perceptual variables (F (1, 26) = 2.54, 0.45, 1.96, and 0.06, respectively; all *p* ≥ 0.122, η²*p* ≤ 0.089).

**Table 2.** Effects of deliberate- and maintenance-practice on physical effort, mental strain, enjoyment, and relevancy.

	Deliberate-practice	Maintenance-practice
**Mental Strain (AU)**	4.18 ± 2.3^#^	1.9 ± 1.2
**Physical Effort (AU)**	3.2 ± 1.5	3.2 ± 1.5
**Enjoyment (AU)**	8.8 ± 1.1	8.4 ± 1.6
**Relevancy (AU)**	7.3 ± 1.3	7.2 ± 1.4

^#^ *Indicates significant differences between deliberate- and maintenance-practice (p < 0.05).*

## 4. Discussion

To the best of our knowledge, the current study is the first to investigate the effects of caffeinated coffee with DP or MP on soccer passing performance in adolescent soccer players. These findings indicate that caffeine intake does not confer an additional advantage on passing technique performance. Regardless of CAF or PLA intake, both DP and MP statistically improve all LSPT values, and MP is the only practice that improves long-pass techniques meaningfully. Interestingly, unlike short passing skills (LSPT), practical affect disappeared for one-touch short passing technique (OTPT). This study also demonstrated that CAF intake may not change practice perceptions. Moreover, when compared to practice regimes without considering CAF or PLA intake, there is no difference between practice activities’ perception except for DP is more mentally compelling than MP.

In line with our results, previous work revealed that moderate doses of caffeine did not have a significant impact on various technical actions [31,34,55–60], in contrast to Del Coso et al. [61] and Perez-Lopez et al. [62], who demonstrated that 3 mg·kg^−1^ of caffeine improves technical actions such as spike and serve in volleyball. Puente et al. [63] also showed that a 3  mg·kg^−1^ dose of caffeine increases total technical performance but not accuracy in free throws and 2–3 point shots in basketball, similar to the findings of Abian Vicen et al. [55]. The ergogenic effect of caffeine may be contingent upon the specific skills being evaluated. Moreover, Fosket et al [32]. found that 6 mg·kg^−1^ of caffeine enhances passing accuracy in soccer, similar to Stuart et al. [64], who found that high doses (6 mg·kg^−1^) of caffeine resulted in a 10% improvement in the ability to pass in rugby players. However, Assi et al. [65] indicated that 6 mg/kg caffeine has no statistical effect on passing accuracy, despite values becoming a higher trend with caffeine ingestion. Dosage may be a determining factor for caffeine's effect on technical skill performance, as higher doses may result in a greater and more prolonged elevation in the blood caffeine concentration [59,66]. In other words, technical aspects of performance could be sensitive to higher caffeine doses. Nevertheless, one must be careful when using higher doses of caffeine due to their possible side effects, such as overarousal, tremors and gastrointestinal discomfort. It would be interesting to investigate the effect of a 6 mg/kg of caffeine dose with different practice activities on soccer passing ability.

The psychostimulant properties of caffeine may provide an alternative explanation for our findings. Caffeine is recognized for its ability to enhance perceptual and cognitive attributes such as alertness, concentration, and reaction time, which are essential for optimizing movement accuracy [67,68] On the other hand, it may also be linked to adverse effects, including nervousness, anxiety, and tremors, potentially impairing task execution, particularly in individuals naive to caffeine ingestion [69]. It can be speculated that the potential side effects and benefits may have counterbalanced one another, as in the famous political phenomenon “Zero-Sum Thinking” [70]. It would be intriguing to examine the effects of caffeine forms with potential reduced side effects (gum or rinses) on skill performance.

While each of the aforementioned explanations could be a reason for our main result, one still must also consider the individual responses to caffeine intake that may vary according to genetic, pharmacological, and environmental factors [71]. One athlete consistently improved all his tests, and six of our participants were still better at short-passing skills and long-pass execution with caffeine intake, despite these differences disappearing at the group level. Moreover, small percentage differences can make big differences on a practical level, even if this has no statistical value. Even a single successful pass might be crucial in a game and influence competitive success. There is an improvement trend in favor of caffeine over 8% for long pass and 2% for short passing technique.

Our study is noteworthy in seeing the effect of DP and MP on short-term technical performance without considering caffeine or placebo intake. Contrary to our expectation, DP is not superior to traditional maintenance repetition in the acute term. One may need to be exposed for a long time if one wants to take advantage of DP’s merits, such as the creation of sophisticated cognitive representations and facilitating long-term memory that underlie skilled performance [72,73]. This is in line with the findings of Shabir et al. [34], who also observed that short-term caffeine ingestion – whether pharmacological or expectancy-based – did not significantly enhance passing performance, suggesting that certain skill-based outcomes may require longer or more complex interventions to be improved. So, it would be interesting to observe the outcomes of implementing both practice types over extended periods. Furthermore, practice conditions such as variability and contextual interference would be another explanation that leads to substantial differences for practice effect [74,75]. However, we relatively create similar practice conditions in terms of variability and contextual interference – except for obscurity in DP-, because this does not violate our DP or MP criteria and not relevant to the purpose of our study.

Importantly, MP is the only practice to increase long pass execution by 22.6%. This development still continues after 48 h (unpublished retention data). Bergman et al. [76] stressed out that “traditional” repetition-based approaches also achieved improvements with respect to players’ technical outcomes, as shown in our study, that traditional rote repetition of movement is still effective for long passes in adolescent soccer players who already develop their actions to a certain extent. On a practice level, coaches may use maintenance long-pass drills in the last training before a game, if developing a technical-tactical plan based on long passes in a game. The observed difference may partly be attributed to the discrepancy in practice duration between DP and MP; however, this possibility appears limited given that the number of repetitions was equal between conditions, and the magnitude and pattern of the practice effects varied across the different passing tasks, indicating practice- or task-specific influences rather than exposure time alone. It is also possible that the slightly higher mental demands of DP induced transient fatigue that may have influenced long-pass execution in the acute term. Nevertheless, DP is still more useful for LSPT values than MP, although it is not statistically different. It suggests that DP may be more effective in opening complex skills than close technical ability in acute terms. This is in line with the findings of Clark et al. [77] showing that 90 min of DP was approximately equivalent to 12 h of unguided practice for complex video game learning in low-performing women.

Finally, this study investigated how athletes perceive practice activities and the effects of caffeine on their perceptions. Although prior studies have demonstrated that caffeine may ameliorate perceived exhaustion, physical–mental stress, and pleasure [59,78–80], this is not evident in the current study. It would be related to the intensity of practice activities; the CAF effect is more pronounced in high-intensity exercises [59,78–80], unlike DP and MP, which were not deemed challenging in this study. It is also worth emphasizing that although DP is considered more mentally challenging than MP, both activities are similar in terms of relevancy, enjoyment, and physicality. Hence, it is necessary to emphasize that even practitioners create the DP environment; athletes’ perceptions could be different from theories in the field. In brief, what one may feel is different from what one felt after he had done. To our knowledge, no study has investigated such a topic before. It would be fascinating to uncover the underlying factors in this theory-action discrepancy in future studies.

This study is not without its limitations. The athletes' diets or calorie intakes were not assessed during the study. In addition, the effectiveness of blinding between the caffeine and placebo conditions was not formally evaluated, and certain perceptual responses were not systematically recorded. The lack of a no-practice control condition should also be taken into account when interpreting the findings, even though a familiarization session was held to minimize any learning effects, and prior research shows that LSPT performance usually stabilizes both within and between days following the initial trials [81,82]. Moreover, given the sample size of the present study, the possibility of Type II error should be acknowledged. Therefore, non-significant findings should be interpreted with caution, and future studies with larger samples are warranted to confirm these results. Furthermore, in developing the DP environment, the focus was on enhancing the non-dominant leg to improve passing proficiency. Nonetheless, non-dominant leg proficiency constitutes merely one potential determinant of overall passing quality. The mechanical assessment of behaviors linked to superior passing skills tailored to each trainee's specific learning requirements could have changed the practice effect. Finally, although LSPT is a widely recognized metric for assessing passing skills, it may fail to replicate competitive performance [83]. Future research should focus on passing metrics in actual match contexts.

## 5. Conclusion

A dose of 3 mg·kg^−1^ of caffeinated coffee does not produce any additional effects on deliberate practice or maintenance practice for passing performance. Regardless of caffeinated coffee or placebo intake, both practices improve short-passing skill, yet only maintenance practice appears effective for enhancing long passing in players with average technical ability. Coaches may consider incorporating these strategies in pre-match warm-ups or structured training programs. Moreover, caffeine intake did not influence players’ perceptions of the training sessions, particularly when physical demand was minimal. When comparing deliberate practice and maintenance practice without considering the caffeine of placebo ingestion – and contrary to our expectations—athletes reported similar perceptions of both approaches. Deliberate practice sessions seem to differ significantly from the original conceptual framework. Therefore, it can be concluded that even when coaches apply deliberate practice criteria on the field, this does not necessarily ensure full alignment with its theoretical definition. The perception of DP may change once it is implemented in real-world settings.

## Supplementary Material

Supplementary Material Supplementary_Material.docx
