# Supplementary material for: Soccer pass performance following caffeine intake with deliberate or maintenance practice
Source: J Int Soc Sports Nutr. 2026 Apr 21;23(1):2663140. doi: 10.1080/15502783.2026.2663140 (PMC13103994; doi:10.1080/15502783.2026.2663140)

***DELIBERATE PRACTICE***

- CONTROL-PASSING DRILLS (40 REPS)

1. Short pass (≤10meters) with non-dominant foot following control (10reps) L

2. Short pass with non-dominant foot following control but in a backward-facing start (10reps) L

3. Short pass with both feet while resolving math questions (10reps) L

4. Short pass with both feet while resolving math questions but in a backward-facing start (10reps) L

- ONE-TOUCH PASSING DRILLS (60 REPS)

1- Short pass with non-dominant foot (10reps) L

2- Short pass with non-dominant foot but in a backward-facing start (10reps) L

3- Short pass to mini-goals utilising dominant foot in accordance with specified colour (10reps) L-D

4- Short pass to mini-goals utilising non-dominant foot in accordance with specified colour (10reps) L-D

5- Short pass to mini-goals utilising dominant foot in foot in accordance with specified colour but in a backward-facing start (10reps) L-D

6- Short pass to mini-goals utilising non-dominant foot in accordance with specified colour but in a backward-facing start (10reps) L-D

- LONG PASS DRILLS (20 REPS)

1- Long pass (≥30 meters) with dominant foot (10reps) L-D

2- Long pass utilising dominant foot in accordance with specified colours but in a backward-facing start (10reps) L-D


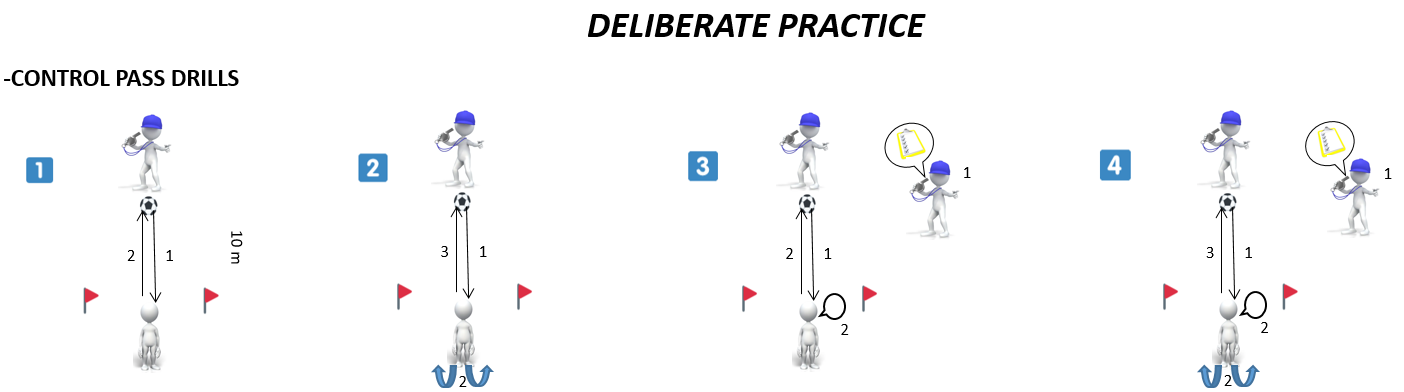


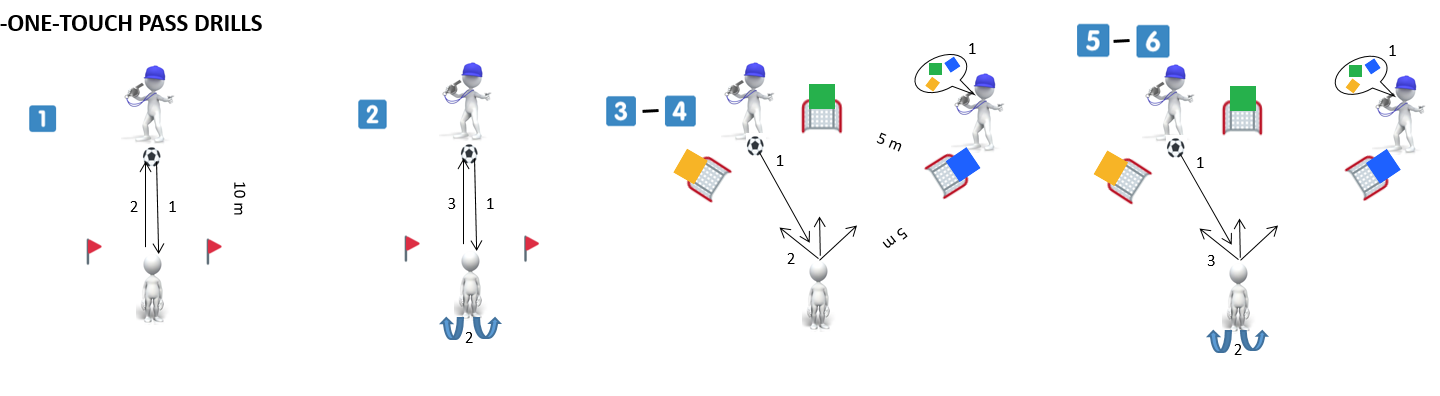


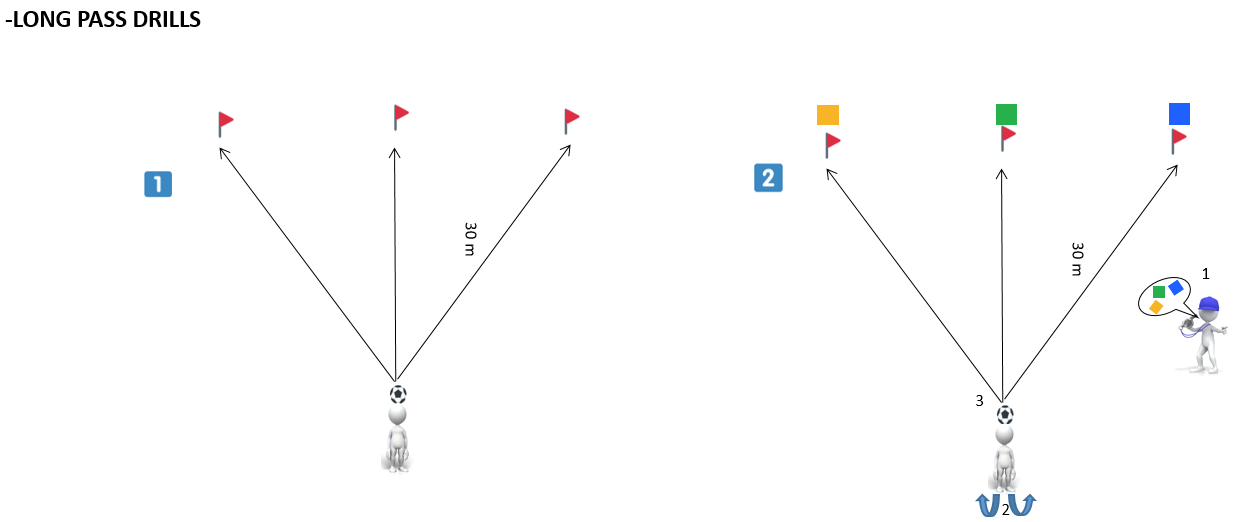


***MAINTENANCE PRACTICE***

- CONTROL-PASSING DRILLS (40 REPS)

1- Two trainees pass (≤10 meters) each other following control in a face-to-face position (40reps) L

- ONE-TOUCH PASSING DRILLS (60 REPS)

1- The trainee executes a one-touch pass to the player positioned in front of  him. Subsequently, manoeuvring around him  and repeating the same movement on the opposite side (20reps) L

2- The trainees perform one-touch diagonal passes to left or right side in groups of four within a quadrangular area. The movement is repeated in reverse way during the next round (40reps) D

- LONG PASSING DRILLS (20 REPS)

1- Two trainees pass (≥30 meters) each other following control (20reps) L-D

L= Linear, D= Diagonal, L-D=Linear/Diagonal


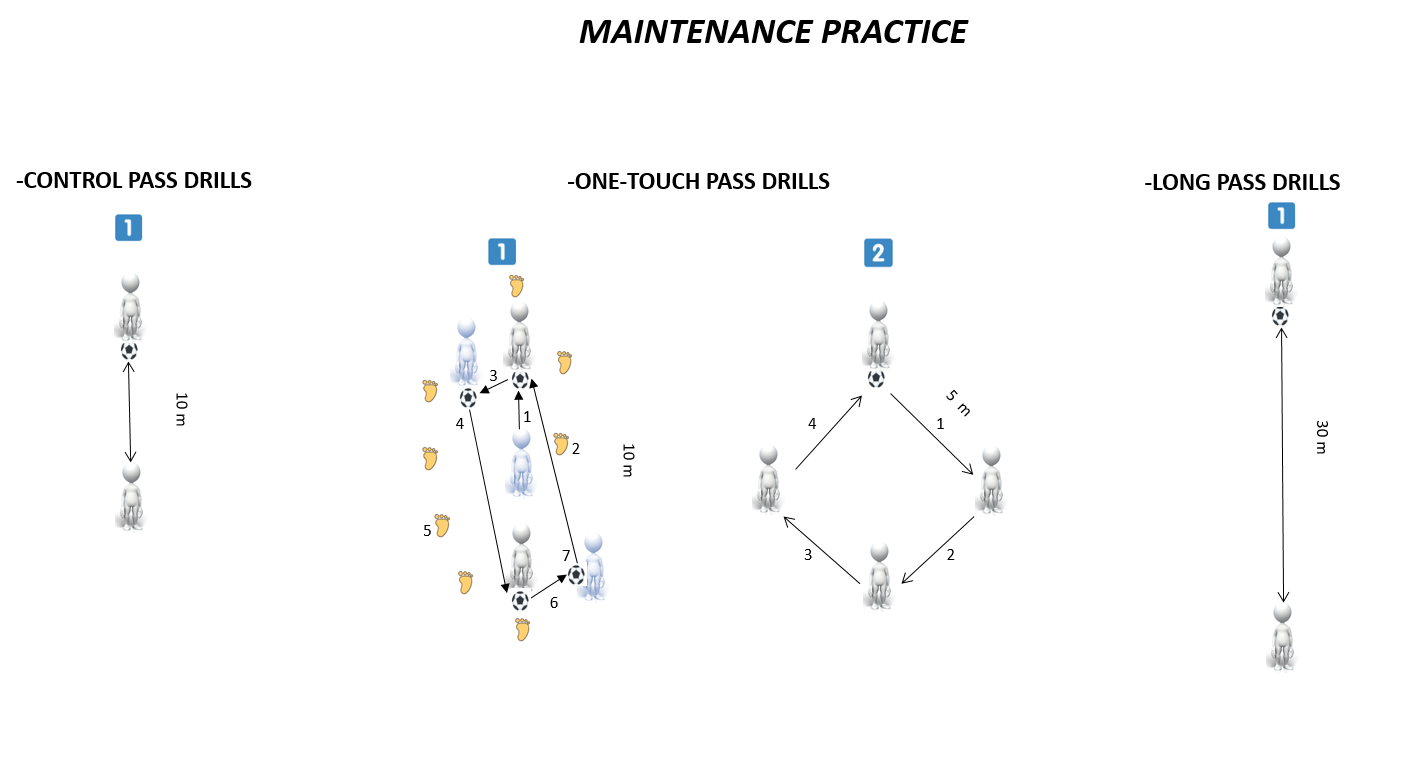

Supplement: Supplementary Material — Supplementary_Material.docx [file RSSN_A_2663140_SM3125.docx]
